# Chemical Composition, Antioxidant, Antimicrobial and Cytotoxic Activities of Essential Oil from *Premna microphylla* Turczaninow

**DOI:** 10.3390/molecules22030381

**Published:** 2017-02-28

**Authors:** Han-Yu Zhang, Yang Gao, Peng-Xiang Lai

**Affiliations:** Marine College, Shandong University, Weihai 264209, China; zhyu19970316@126.com (H.-Y.Z.); 18813297064@163.com (Y.G.)

**Keywords:** *Premna microphylla* Turczaninow, essential oil, GC-MS, antioxidant activity, antibacterial activity, antifungal activity, cytotoxic activity

## Abstract

*Premna microphylla* Turczaninow*,* an erect shrub, was widely used in Chinese traditional medicine to treat dysentery, appendicitis, and infections. In this study, the essential oil from *P. microphylla* Turcz. was obtained by hydrodistillation and analyzed by GC (Gas Chromatography) and GC-MS (Gas Chromatography-Mass Spectrometer). Fifty-six compounds were identified in the oil which comprised about 97.2% of the total composition of the oil. Major components of the oil were blumenol C (49.7%), β-cedrene (6.1%), limonene (3.8%), α-guaiene (3.3%), cryptone (3.1%), and α-cyperone (2.7%). Furthermore, we assessed the in vitro biological activities displayed by the oil obtained from the aerial parts of *P. microphylla*, namely the antioxidant, antimicrobial, and cytotoxic activities. The antioxidant activity of the essential oil was evaluated by 1,1-diphenyl-2-picrylhydrazyl (DPPH) radical scavenging activity. For this, the IC_50_ value was estimated to be 0.451 mg/mL. The essential oil of *P. microphylla* exhibited considerable antibacterial capacity against *Escherichia coli* with an MIC (Minimum Inhibitory Concentration) value of 0.15 mg/mL, along with noticeable antibacterial ability against *Bacillus subtilis* and *Staphylococcus aureus* with an MIC value of 0.27 mg/mL. However, the essential oil did not show significant activity against fungus. The oil was tested for its cytotoxic activity towards HepG2 (liver hepatocellular cells) and MCF-7 Cells (human breast adenocarcinoma cell line) using the MTT (3-(4,5-dimethyl-2-thiazolyl)-2,5-diphenyl-2-H-tetrazolium bromide) assay, and exerted cytotoxic activity with an IC_50_ of 0.072 and 0.188 mg/mL for 72 h. In conclusion, the essential oil from *P. microphylla* is an inexpensive but favorable resource with strong antibacterial capacity as well as cytotoxic activity. Thus, it has the potential for utilization in the cosmetics and pharmaceutical industries.

## 1. Introduction

The genus *Premna* (Verbenaceae), widely distributed in tropical and subtropical areas such as Asia, Africa, Australia, and the Pacific Islands, is an important medicinal plant family [1]. This genus consists of more than 200 species in the world, of which about 45 species are distributed in China. They have been found to display a wide spectrum of pharmacological effects involving anticancer, antibacterial, antifungal, and anti-leishmania activities [2]. *P. microphylla* Turcz., a deciduous shrub, belongs to this genus. It is broadly distributed in the mountainous regions in the east, middle, and south of China. Leaves of *P. microphylla* are widely used to treat dysentery, appendicitis, and infections [3].

Antioxidants can inhibit or delay the oxidation of oxidizable substrates, and this appears to be very important in the prevention of oxidative stress, which is suggested as one of the leading causes of many oxidation-related diseases [4]. Resistance against antibiotics by pathogenic bacteria is a major concern in the anti-infective therapy of both humans and animals [5]. Recently, many consumers prefer additive free foods or a safer approach like the utilization of more effective antioxidant and antibacterial agents from natural origins. Plant essential oils and their components have been shown to possess multiple and varied biological activities such as antimicrobial, cytotoxic, and antioxidant activities [6].

There were no literature reports on the chemical composition and biological activities of the essential oil from *P. microphylla.* Therefore, we used the hydrodistillation method to collect the oil, and we identified the chemical composition of the oil by GC-FID (Gas Chromatography-Flame Ionization Detection) and GC-MS (Gas Chromatography-Mass Spectrometer). Furthermore, the essential oil was investigated for its antioxidant, antimicrobial, and cytotoxic activities.

## 2. Results

### 2.1. Chemical Composition

The components of the essential oil with their retention indices (RI) from the aerial parts of *P. microphylla* are listed in Table 1. GC-MS analyses revealed that the essential oil had a complex chemical profile. Separation of volatile components was accomplished by using a polar column (HP-5 MS). A total of fifty-six compounds represented 97.2% of the oil. The main fraction of the essential oil consisted of monoterpenes (60.6%), represented by oxygenated compounds (56.4%) and hydrocarbons (4.2%). The essential oil composition was dominated by blumenol C (49.7%), β-cedrene (6.1%), limonene (3.8%), α-guaiene (3.3%), cryptone (3.1%), and α-cyperone (2.7%).

### 2.2. Antioxidant Activity

The DPPH test aimed to measure the capacity of the essential oil to scavenge the free radical 1,1-diphenyl-2-picrylhydrazyl (DPPH) by the donation of hydrogen atoms or electrons [8]. The effect of the oil on DPPH radical scavenging was compared to BHT (Butylated Hydroxytoluene), used as positive control, and the IC_50_ values were determined. The scavenging effect of the essential oil and standard BHT on the DPPH radical expressed as IC_50_ values was 0.451 mg/mL for *P. microphylla* vs. 0.067 mg/mL for BHT (Table 2). Therefore, the oil did not show a high antioxidant activity compared to the BHT, as presented in Table 2.

### 2.3. Antimicrobial Activity

The antimicrobial activities of the essential oil from *P. microphylla* were qualitatively and quantitatively assessed by the presence or absence of inhibition zone diameters (IZD) and MIC (Minimum Inhibitory Concentration) values by the microdilution method. The results are presented in Table 3. The inhibition zone diameter of almost 15 mm was indicative of a good antimicrobial activity. Additionally, the corresponding minimum inhibitory concentration (MIC) was equal to 0.512 mg/mL [9]. Thus, the essential oil of *P. microphylla* showed a strong antibacterial activity against the tested Gram-positive and negative bacteria with MIC values of 0.270 mg/mL (IZD: 15.1–15.4 mm) and 0.150–0.480 mg/mL (IZD: 9.0–21.8 mm), respectively. The best activities were observed against *E. coli* with an MIC value of 0.150 mg/mL (IZD: 21.8 mm) followed by *B. subtilis, S. aureus*, and *P. aeruginosa*. However, it did not have a significant activity against the fungus (MIC: >0.5 mg/mL).

### 2.4. Cytotoxic Activity

General cell toxic effects of *P. microphylla* essential oil were evaluated by using the MTT assay against HepG2 cells (liver hepatocellular cell line) and MCF-7 cells (human breast adenocarcinoma cell line). All cell lines were subjected to increasing concentrations of essential oil for 24 h, 48 h, and 72 h. The results are shown in Table 4 and Table 5. The essential oil exhibited a significant growth inhibition against the examined human cancer cells and induced a concentration-dependent inhibitory effect at concentrations ranging from 0.016–2 mg/mL. The oil was found to be able to inhibit HepG2 cells (liver hepatocellular cells), with an IC_50_ of 0.072 mg/mL (Table 4), as well as MCF-7 cells (human breast adenocarcinoma cell line), with an IC_50_ of 0.188 mg/mL (Table 5) after 72 h of incubation. 

## 3. Discussion

To our knowledge, there are no literature reports on the chemical composition and biological activities of the essential oil from *P. microphylla.* Thus, our findings are of interest. In this study, the oxygenated monoterpenes (56.4%) were revealed as predominant over the monoterpene hydrocarbons (4.2%). The oil was found to contain primarily blumenol C (49.7%), β-cedrene (6.1%), limonene (3.8%), α-guaiene (3.3%), cryptone (3.1%), and α-cyperone (2.7%). Blumenol C was first isolated from the leaves of *Podocarpus blumei* in 1972. The absolute stereochemistry of blumenol C was then determined as (6*R*,9*R*) by the chemical conversion of a related compound. Its glucoside, i.e., blumenol C glucoside, was then isolated from the aerial parts of *Epimedium grandiflorum* var. *thunbergianum* [10]. β-Cedrene is a bioactive sesquiterpene which exerts antiseptic, anti-inflammatory, antispasmodic, tonic, astringent, diuretic, sedative, insecticidal, and antifungal activities [11]. This compound is used in traditional medicine and in cosmetic products. Moreover, cedrene is found to be a potent competitive inhibitor of CYP2B6-mediated bupropion hydroxylase [12]. Another main component is limonene, which is common in cosmetic products. As a flavouring compound, limonene is used in food manufacturing and certain medicines, e.g., as a flavoring to mask the bitter taste of alkaloids. In natural and alternative medicine, it is marketed to relieve gastroesophageal reflux disease and heartburn [13]. Additionally, limonene is being increasingly used as a solvent for cleaning purposes, such as the removal of oil from machines [14].

In the radical scavenging activity assay, the essential oil of *P. microphylla* displayed weak activity. Essential oils are quite complex mixtures composed of a great variety of compounds. Generally, because of this complexity it is difficult to explain their activities. Many reports on the antioxidant potentials of essential oils often refer to concepts such as synergism, antagonism, and additivity [15]. The chemical components of the oil were dominated by blumenol C (49.7%), β-cedrene (6.1%), and limonene (3.8%), which displayed low inhibition of free radicals. The antioxidant property of the *Eucalyptus globulus* essential oil stands out when compared to the *P. microphylla* oil, since its IC_50_ value is 0.065 mg/mL [16]. It could be inferred that the antioxidant activity of the *Eucalyptus globulus* oil is mainly due to the presence of its major compounds, namely 1,8-cineole (63.8%). 1,8-cineole showed various degrees of reducing power, radical scavenging, chelating, in addition to its DNA-protective capacity [17]. Moreover, the high value of the reducing power indicated that the essential oil components are able to act as electron donors and reduce the oxidized intermediate of lipid peroxidation so that they can act as primary and secondary antioxidants [18].

The results from the growth inhibition zone diameters (IZD) and the MIC values indicated that the essential oil from *P. microphylla* had strong inhibitory effects against all Gram-negative bacteria and certain Gram-positive bacteria, with MIC values of 0.270 mg/mL (IZD:15.1–15.4 mm) and 0.150 mg/mL (IZD: 9.0–21.8 mm), respectively. *E. coli* was the most sensitive strain with an MIC value of 0.150 mg/mL (IZD: 21.8 mm) followed by *B. subtilis* and *S. aureus* with an MIC value of 0.270 mg/mL (IZD: 15.4 mm). However, it did not have significant activity against *P. aeruginosa*, along with an absence of antifungal activity, since *P. aeruginosa* possesses an intrinsic resistance, which is associated with the nature of its outer membrane to a wide range of biocides. The outer layer of the Gram-negative outer membrane is composed primarily of lipopolysaccharide. This forms a hydrophilic permeability barrier which provides protection against the effects of toxic agents [19]. These compounds (except terpinen-4-ol) are normally inactive because they are unable to effectively penetrate the outer membrane [20], thus the presence of a low concentration of terpinen-4-ol (0.7%) leads to the absence of significant effects on *P. aeruginosa.* It is well known that essential oils and their components show a better antimicrobial effectiveness against Gram-positive bacteria. Due to the composition of the outer membrane, essential oil can not only alter such structures but can penetrate within the cell, leading to alterations such as the denaturation of proteins and enzymes, the “unbalance” of the K^+^ and H^+^ ion concentration, to the modification of the entire cell morphology, which can lead to the death of the microorganism [21,22,23].

Phenolic compounds generally show a good antimicrobial effectiveness against Gram-positive bacteria; their effect is dependent on their amount: at low concentrations, they are able to interfere with enzymes involved in the production of energy; at higher concentrations, they can induce the denaturation of proteins until an irreversible modification of the cell and death. Thus, the strong antibacterial activity of the essential oil of *P. microphylla* against the tested bacteria could be attributed to the presence of the high concentration of blumenol C (49.7%) [24].

The microorganisms tested in the present investigation are large and cover the most important human pathogens known as opportunists for man and animals, which cause food contamination and deterioration. The obtained results are significant, particularly in the case of *E. coli* which is well-known for causing gastroenteritis, urinary tract infections, and neonatal meningitis [25]. The results above indicated that the oil was noticeably active and may be useful in pharmaceutical engineering. 

This work reports on the cytotoxic activity of *P. microphylla* essential oil. It shows cytotoxic activities on breast adenocarcinoma MCF-7 (0.188 mg/mL, IC_50_) and hepatocarcinoma HepG2 cells (0.072 mg/mL, IC_50_). The results quite modestly display that the oil does affect the growth of these tested cancer cell lines. From this, certain antitumor activity can be expected but remains to be further explored. The toxic activity of the essential oil of *P. microphylla* against the tested bacteria could be attributed to the presence of significant amounts of limonene (3.8%), β-cedrene (6.1%), α-cyperone (2.7%), and myristicin (1.2%). In previous studies, the principal metabolites of limonene are *trans*-carveol, a product of 6-hydroxylation, and perillyl alcohol, a product of 7-hydroxylation by the CYP2C9 and CYP2C19 cytochromes in human liver microsomes [26]. Perillyl alcohol has been investigated for its pharmacological activity as a dietary chemotherapeutic agent. They are viewed as novel therapeutic options in some CNS neoplasms and other solid tumours, particularly for the treatment of gliomas [27]. The cytotoxic activities of perillyl alcohol and limonene metabolites are likely due to their antiangiogenic activities, hyperthermia inducing effects, as well as negative apoptosis regulation and Ras (Renin-Agiotensin System) pathways [28]. Moreover, β-cedrene is considered to be a powerful antimicrobial and antitumor agent [29]. α-Cyperone has been shown to alleviate lung cell injury caused by *Staphylococcus aureus*, to be effective against *E.* *coli*, and to act as an anti-inflammatory agent [30,31,32]. Myristicin is a naturally occurring alkenylbenzene compound found in nutmeg, and it induces cytotoxicity in SK-N-SH cells by an apoptotic mechanism [33]. Moreover, besides the contribution of specific constituents, the synergism between various components of the essential oil could play an important role in the cytotoxic effect verified in this study against HepG2 and MCF-7 cells.

## 4. Experimental Section

### 4.1. Plant Material

*P. microphylla* was collected from the Zhejiang Province of China in September 2016, and was identified by Associate Prof. Zhao of Shandong University (Weihai, China). A voucher specimen (No. 1703) was deposited at the Laboratory of Botany of Marine College, Shandong University.

### 4.2. Essential Oil Isolation

For the plant material, the aerial part of *P. microphylla* (500 g) was hydrodistilled for six hours by using a Clevenger apparatus to obtain the essential oil (1.56 g, 0.31% *w/w*), and the oil was stored at 4 °C until analysis.

### 4.3. Gas Chromatography Analysis 

The oil sample was analyzed by using an Agilent 6890 gas chromatograph (Agilent, Santa Clara, CA, USA) equipped with flame ionization detector (FID, Agilent). Column: capillary column HP-5MS, 30 m × 0.25 mm × 0.25 μm film thickness. The GC oven temperature was programmed to stay at 60 °C for 1 min, heat to 200 °C at a rate of 15 °C/min, keep constant at 200 °C for 5 min, and then heat to 280 °C at a rate of 5 °C/min, and keep constant at 280 °C for 2 min [34]. 

### 4.4. Gas Chromatography-Mass Spectrometry

GC-MS analyses were carried out using a Hewlett Packard 6890 gas chromatograph (Agilent) fitted with an HP-5MS fused silica column, interfaced with a Hewlett Packard 5975C mass selective detector operated by HP Enhanced ChemStation software (Agilent). The oven temperature was programmed from 60–280 °C at 5 °C/min using helium as a carrier gas at 1.2 mL/min. The injector temperature was 250 °C; the injection volume was 0.2 μL of 1% solution prepared in n-hexane; split ratio 1:50. GC parameters were the same as above; Mass spectra were recorded at 70 eV. The mass range was from *m*/*z* 50 to 550.

### 4.5. Identification of Components

The oil components were identified from their GC retention indices (RI) obtained with reference to n-alkanes series on a HP-5MS column, and comparison of their mass spectra and fragmentation patterns by computer matching with the Nist 8.0 Mass Spectral Database for GC-MS. The relative amounts of individual components were calculated based on GC peak area (FID response) without using a correction factor. The result of the analysis is shown in Table 1.

### 4.6. Antioxidant Activity Assay

The antioxidant activity of the essential oil was determined with the DPPH radical protocol [35]. DPPH is a well-known radical and a trap for other radicals. Therefore, the rate reduction of a chemical reaction upon addition of DPPH is used as an indicator of the radical nature of that reaction. Because of a strong absorption band centered at about 520 nm, the DPPH radical has a deep violet color in solution, and it becomes colorless or pale yellow when neutralized. This property allows visual monitoring of the reaction, and the number of initial radicals can be counted from the change in the optical absorption at 520 nm or in the EPR (Electron Spin Resonance) signal of the DPPH [36]. Free radical scavenging activity of the essential oil was measured by a spectrophotometer at 517 nm. A methanol solution of DPPH was prepared immediately before the assay. Various concentrations of each 2 mL sample (0.100, 0.150, 0.200, 0.250, 0.300, 0.350, 0.400, 0.450, 0.500 mg/mL) were added to 2 mL of DPPH solution. The reaction mixture was shaken and allowed to stand for 30 min in the shade. The absorbance of the samples was measured by a spectrophotometer at 517 nm and calculated by the following equation:

DPPH radical scavenging activity = (A_0_ − A_1_)/A_0_ × 100% (1)

where A_0_ is the absorbance of the control (blank, without extract) and A_1_ is the absorbance of the mixture with the essential oil. In this assay, butylated hydroxytoluene (BHT) was used as a standard antioxidant to validate the assay. The experiment was repeated three times. The result of the antioxidant activity test is shown in Table 2.

### 4.7. Antimicrobial Activity

#### 4.7.1. Bacterial and Fungal Strains

Four bacteria strains were selected for the antibacterial tests, including the Gram-positive bacteria *Staphylococcus aureus* (ATCC 6538) and *Bacillus subtilis* (ATCC 6633), and the Gram-negative bacteria *Escherichia coli* (ATCC 25922) and *Pseudomonas aeruginosa* (ATCC 27853). 

The following fungal strains were also tested: *Candida albicans* (ATCC 10231), *Aspergillus niger* (ATCC 16404), and *Fusarium oxysporum* (ATCC 695).

#### 4.7.2. Agar Diffusion Methods

The antibacterial and antifungal activities of the essential oil of *P. microphylla* were assessed using paper disk agar diffusion, according to the method described by the National Committee for Clinical Laboratory Standard, with some modification [37]. Absorbent disks (Whatman disk of 6 mm diameter) were impregnated with 15 μL of essential oil or chloramphenicol, ampicillin, or ketoconazole as a positive control and were incubated at 37 °C for 18 h. Antimicrobial activity was assessed by measuring the inhibition zone, by examining the disk diffusion plates from the back, viewed against a black background and illuminated with reflected light. With a caliper, the diameter of each zone of inhibition was measured and recorded to the nearest whole millimeter. This is the diameter of the zone visibly presenting the absence of bacterial growth, including the 6 mm disk.

#### 4.7.3. Determination of the Minimum Inhibition Concentration (MIC)

The minimum inhibitory concentration (MIC) values were determined for all oils by using the microplate dilution method [38]. Stock solutions of the oils and the standard antibacterial compound chloramphenicol were prepared in the Mueller Hinton Broth liquid medium, and stock solutions of the oils and standard antifungal compounds ampicillin and ketoconazole were prepared in the Potato Dextrose Agar (PDA) liquid medium. Serial dilution of the initial concentrations were prepared on 96-well microlitre plates containing equal amounts of distilled water. The microbial suspension concentration was standardized to McFarland No. 0.5 after incubation for 24 h at 37 °C. Cultures were mixed with essential oils and were incubated for 24 h at 37 °C. The minimum inhibitory concentration (MIC: mg/mL) was detected as the minimum concentration where microbial growth was missing. 1% 2,3,5-Triphenyltetrazolium chloride (TTC) was used as an indicator of microbial growth. In order to facilitate the dispersion of the essential oil in the aqueous nutrient medium, it was diluted with Tween 20, at a ratio of 15%. The negative control containing only Tween 20 was not toxic to the microorganisms [39,40]. Chloramphenicol, ampicillin, and ketoconazole were used as positive controls. All the experiments were performed in triplicate. The results of the bacterial activity tests are given in Table 3.

### 4.8. Cytotoxic Activity

HepG2 (liver hepatocellular cells) and MCF-7 cells (human breast adenocarcinoma cell line) were cultured in a humidified atmosphere of 5% CO_2_ in air at 37 °C, in RPMI1640 medium containing 10% (*v*/*v*) fetal calf serum, 2 mM glutamine, and antibiotics (200 U of penicillin and 50 mg of streptomycin per liter) and were maintained at 37 °C in a humidified 5% CO_2_ atmosphere [41]. Cytotoxicity was measured using the MTT test with slight modifications. Cells were seeded at 5 × 10^3^ cells/well in 200 μL of growth medium and were incubated at 37 °C for 24 h for cell adherence. The microplates were treated with the substances and incubated for 24, 48, and 72 h.

Then, 10 μL of MTT was added to each well (5 mg/mL) and the incubation was continued for a further 2 h. 100 μL of DMSO was added to each well. The absorbance (A) was measured at 570 nm using a Multiskan Ascent microplate reader (Thermo Fisher Scientific, Waltham, MA, USA). The assay was realized in triplicate as a cell viability index. The percentages of cell growth were calculated as follows:

Cell growth (%) = [A (sample)/A (control)] × 100%(2)

Cytotoxicity is expressed as the concentration of extract inhibiting cell growth by 50% (IC_50_) [42]. The results of the cytotoxicity activity tests are given in Table 4 and Table 5.

## 5. Conclusions

Due to no previous knowledge, we identified the chemical components of the essential oil from *P. microphylla* by using GC-MS. Furthermore, we studied its antioxidant, antimicrobial, and cytotoxic activities. The oil was shown to possess high levels of oxygenated monoterpenes with strong antibacterial and antitumor potential which might be due to the presence of blumenol C (49.7%), cedrene (6.1%), and limonene (3.8%), potentially leading to its ecofriendly and beneficial utilization in pharmaceutical applications.

Our research provide evidence that the aerial parts of *P. microphylla* can be considered as a good source of bioactive compounds such as blumenol C. Moreover, the results indicate that the oil from *P. microphylla* exhibits antibacterial activity against *Escherichia coli* with an MIC value of 0.15 mg/mL, and also some antibacterial activity against *Bacillus subtilis* and *Staphylococcus aureus* with an MIC value of 0.27 mg/mL. It may thus have a certain potential to cure gastroenteritis, urinary tract infections, and neonatal meningitis. 

## Figures and Tables

**Table 1:** Chemical composition of the essential oil of *Premna microphylla* Turcz.

Peak No.	Compound ^a^	RI ^b^	RI ^c^	Peak Area %	Identification ^d^
1	δ-3-Carene	1007	1007	0.2	MS, RI
2	Limonene	1031	1031	3.8	MS, RI
3	p-Cymene	1095	1095	0.3	MS, RI
4	α-Campholenal	1132	1131	0.2	MS, RI
5	*cis*-Verbenol	1146	1144	0.1	MS, RI
6	Terpinen-4-ol	1185	1187	0.7	MS, RI
7	Cryptone	1195	1192	3.1	MS, RI
8	Verbenone	1219	1219	0.4	MS, RI
9	Cuminal	1249	1248	1.0	MS, RI
10	Piperitone	1262	1262	0.2	MS, RI
11	Neral	1266	1268	0.3	MS, RI
12	Phellandral	1286	1281	0.2	MS, RI
13	Perillaldehyde	1295	1286	0.2	MS, RI
14	Piperitenone	1306	1309	0.5	MS, RI
15	α-Copaene	1389	1390	0.2	MS, RI
16	β-Elemene	1403	1403	1.4	MS, RI
17	α-Cedrene	1433	1432	1.2	MS, RI
18	β-Cedrene	1442	1446	6.1	MS, RI
19	α-Himachalene	1459	1460	0.2	MS, RI
20	γ-Himachalene	1470	1468	0.3	MS, RI
21	Ledene	1475	1473	0.1	MS, RI
22	α-Guaiene	1482	1482	3.3	MS, RI
23	Eremophilene	1504	1502	1.3	MS, RI
24	β-Selinene	1514	1509	1.0	MS, RI
25	Myristicin	1532	1529	1.2	MS, RI
26	Spathulenol	1550	1548	1.0	MS, RI
27	Elemicin	1558	1558	1.2	MS, RI
28	Geranyl butyrate	1574	1570	0.4	MS, RI
29	α-Cedrol	1581	1580	0.2	MS, RI
30	Caryophyllene oxide	1601	1601	1.5	MS, RI
31	Epiglobulol	1605	1608	0.3	MS, RI
32	Epicedrol	1630	1632	0.9	MS, RI
33	Acorenone B	1638	1632	0.4	MS, RI
34	Widdrol	1651	1651	0.4	MS, RI
35	2-Methylhexadecane	1658	1665	0.3	MS, RI
36	Bulnesol	1666	1666	0.5	MS, RI
37	α-Cedrenol	1673	1669	0.7	MS, RI
38	Farnesol *	1684	1682	0.4	MS, RI
39	*cis*-14-nor-Muurol-5-en-4-one	1700	1696	2.4	MS, RI
40	Blumenol C	1715	1713	49.7	MS, RI
41	α-Cyperone	1748	1755	2.7	MS, RI
42	Cedranyl acetate	1787	1780	0.5	MS, RI
43	Saussurea lactone	1803	1806	0.2	MS, RI
44	Nootkatone	1810	1810	0.2	MS, RI
45	Dehydrofukinone	1823	1820	0.2	MS, RI
46	Neophytadiene	1832	1836	0.4	MS, RI
47	Perhydrofarnesyl acetone	1839	1836	0.2	MS, RI
48	Corymbolone	1892	1898	0.2	MS, RI
49	β-Cyclodihydrocostunolide	1947	1947	0.1	MS, RI
50	Kaurene	2057	2061	0.7	MS, RI
51	1-Octadecanol	2095	2090	0.1	MS, RI
52	Oleic Acid	2139	2140	1.1	MS, RI
53	Octadecyl acetate	2203	2205	1.1	MS, RI
54	Eicosanol	2299	2290	0.8	MS, RI
55	Docosanoic methyl ester	2523	2524	0.2	MS, RI
56	Methyl tetracosanoate	2726	2725	0.7	MS, RI
	monoterpene hydrocarbons			4.2	
	oxygenated monoterpenes			56.4	
	sesquiterpene hydrocarbons			14.8	
	oxygenated sesquiterpenes			13.8	
	diterpenes			1.1	
	Total			97.2	

Notes: Compounds ^a^ are listed in order of their elution from a HP-5MS column; RI ^b^ (retention index): RI-non-isothermal Kovats retention indices on a HP-5MS column; RI ^c^ linear retention indices from the literature (NIST 08 Mass Spectra Library (Version 2.0 f) and WILEY’S Library of Mass spectra 9th Edition [7]) on a HP-5MS column; Identification ^d^: RI: Linear Retention index; MS: Mass Spectrometry; * correct isomer not identified.

**Table 2:** DPPH scavenging activity of the essential oil of *Premna microphylla* Turcz.

Concentration (mg/mL)	DPPH Scavenging Activity ^a^	
	*P. microphylla*	BHT ^b^
0.10	15.5 ± 2.0	71.5 ± 5.3
0.15	19.9 ± 1.8	85.7 ± 3.3
0.20	25.7 ± 2.2	90.3 ± 0.8
0.25	31.7 ± 2.3	91.7 ± 1.1
0.30	36.5 ± 1.0	92.5 ± 2.3
0.35	46.4 ± 2.8	94.1 ± 0.8
0.40	47.1 ± 1.7	95.7 ± 0.9
0.45	48.6 ± 3.5	96.5 ± 0.1
0.50	52.3 ± 1.2	98.7 ± 0.4

Antioxidant activity ^a^ (inhibition %), expressed as the mean ± SD of triplicate experiments. Positive control ^b^ used.

**Table 3:** Antibacterial and antifungal activity of the essential oil of *Premna microphylla* Turcz.

Microorganism	Diameter of the Inhibition Zones (mm) ^a^				MIC (mg/mL)			
	EO	Ch	Am	Ke	EO	Ch	Am	Ke
**Gram positive**								
*Staphylococcus aureus* ATCC 6538	15.1 ± 0.5	27.8 ± 0.9	N.T.	N.T.	0.270	0.025	N.T.	N.T.
*Bacillus subtilis* ATCC 6633	15.4 ± 0.2	30.2 ± 1.0	N.T.	N.T.	0.270	0.013	N.T.	N.T.
**Gram negative**								
*Escherichia coli* ATCC 25922	21.8 ± 0.4	32.5 ± 0.5	N.T.	N.T.	0.150	0.125	N.T.	N.T.
*Pseudomonas aeruginosa*								
ATCC 27853	9.0 ± 0.4	15.5 ± 0.4	N.T.	N.T.	0.480	0.100	N.T.	N.T.
**Fungus**								
*Candida albicans* ATCC 10231	7.0 ± 0.2	N.T.	35.5 ± 0.8	N.T.	>0.500	N.T.	0.005	N.T.
*Aspergillus niger* ATCC 16404	6.9 ± 0.8	N.T.	N.T.	32.4 ± 0.4	>0.500	N.T.	N.T.	0.003
*Fusarium oxysporum* ATCC 695	6.8 ± 0.7	N.T.	N.T.	31.8 ± 0.4	>0.500	N.T.	N.T.	0.003

The diameter of the inhibition zones (mm), including the disc diameter (6 mm), are given as the mean ± SD of triplicate experiments. Diameter of the inhibition zones ^a^ of the EO: essential oil of *P. microphylla* (tested volume, 1 mg/mL); positive control: Ch, chloramphenicol; Am, ampicillin; Ke, ketoconazole (tested volume, 0.01 mg/mL); N.T.: not tested.

**Table 4:** Cytotoxic activity of the essential oil from *Premna microphylla* Turcz. against HepG2 Cells (liver hepatocellular cells).

Concentration (mg/mL)		0.016	0.031	0.063	0.125	0.250	0.500	1	2	IC_50_ (mg/mL) ^a^
Cell growth (%)	24 h	96.2	95.5	99.9	98.0	90.3	91.9	97.3	73.4	>2
	48 h	95.3	96.8	89.3	79.3	56.7	31.2	16.0	6.4	0.315 ± 0.023
	72 h	68.8	57.4	56.2	42.3	39.0	20.0	11.3	7.5	0.072 ± 0.005
Cisplatin ^b^										0.003 ± 0.001

IC_50_^a^ = the concentration of compound that affords a 50% reduction in cell growth (after 24, 48, and 72 h of incubation); Cisplatin ^b^ was tested as a reference; Expressed as the mean ± SD of triplicate experiments.

**Table 5:** Cytotoxic activity of the essential oil from *Premna microphylla* Turcz. against MCF-7 Cells (human breast adenocarcinoma cell line).

Concentration (mg/mL)		0.016	0.031	0.063	0.125	0.250	0.500	1	2	IC_50_^a^ (mg/mL)
Cell growth (%)	24 h	98.4	99.7	97.6	96.5	86.9	82.7	80.2	64.7	>2
	48 h	98.5	95.3	94.2	92.6	91.9	73.3	34.4	6.5	0.751 ± 0.045
	72 h	99.0	85.9	82.3	64.6	45.3	14.4	4.4	3.6	0.188 ± 0.021
Cisplatin ^b^										0.003 ± 0.01

IC_50_^a^ = the concentration of a compound that affords a 50% reduction in cell growth (after 24, 48, and 72 h of incubation); Cisplatin ^b^ was tested as a reference; Expressed as the mean ± SD of triplicate experiments.

## References

[1] Ayinampudi S.R., Domala R. (2012). New icetexane diterpenes with intestinal α-glucosidase inhibitory and free-radical scavenging activity isolated from *Premna tomentosa*, roots. Fitoterapia.

[2] Suresh G., Babu K.S. (2011). Novel cytotoxic icetexane diterpenes from *Premna latifolia* Roxb. Tetrahedron Lett..

[3] Zhong C.G., Wang D.Y. (2002). Four new isoflavones from *Premna microphylla*. Indian J. Heterocycl. Chem..

[4] Kemper N. (2008). Veterinary antibiotics in the aquatic and terrestrial environment. Ecol. Indic..

[5] Ben H.A., Hamdi N. (2013). Characterization of essential oil from *Citrus aurantium* L flowers: Antimicrobial and antioxidant activities. J. Oleo Sci..

[6] Bakkali F., Averbeck S. (2008). Biological effects of essential oils—A review. Food Chem. Toxicol.,.

[7] Hammami S., Jmii H. (2015). Essential oil composition, antioxidant, cytotoxic and antiviral activities of *Teucrium pseudochamaepitys* growing spontaneously in Tunisia. Molecules.

[8] Tepe B., Daferera D. (2005). Antimicrobial and antioxidant activities of the essential oil and various extracts of *Salvia tomentosa*, Miller (Lamiaceae). Food Chem..

[9] Santos N., Pascon R. (2016). Cytotoxic and antimicrobial constituents from the essential oil of *Lippia alba* (Verbenaceae). Medicines.

[10] Matsunami K., Otsuka H. (2010). Structural revisions of blumenol C glucoside and byzantionoside B. Chem. Pharm. Bull..

[11] Jeong H.U., Kwon S.S. (2014). Inhibitory effects of cedrol, β-cedrene, and thujopsene on cytochrome p450 enzyme activities in human liver microsomes. J. Toxicol. Environ. Health A.

[12] Oh I., Yang W.Y. (2011). In vitro Na^+^/K^+^-ATPase inhibitory activity and antimicrobial activity of sesquiterpenes isolated from *Thujopsis dolabrata*. Arch. Pharm. Res..

[13] Sun J. (2007). d-limonene: Safety and clinical applications. Altern. Med. Rev. A J. Clin. Ther..

[14] Pike J.A., Dorricott W.J. (1999). Self-Adhesive Stamps. U.S. Patent.

[15] Ben H.A., Ben H.N. (2013). Essential oil from *Artemisia phaeolepis*: Chemical composition and antimicrobial activities. J. Oleo Sci..

[16] Luís Â., Duarte A., Gominho J. (2016). Chemical composition, antioxidant, antibacterial and anti-quorum sensing activities of *Eucalyptus globulus*, and *Eucalyptus radiata*, essential oils. Ind. Crop Prod..

[17] Horvathova E., Navarova J. (2014). Assessment of antioxidative, chelating, and dna-protective effects of selected essential oil components (eugenol, carvacrol, thymol, borneol, eucalyptol) of plants and intact *Rosmarinus officinalis* oil. J. Agric. Food Chem..

[18] Petrelli R., Orsomando G. (2016). Biological activities of the essential oil from *Erigeron floribundus*. Molecules.

[19] Nikaido H. (1985). Molecular basis of bacterial outer membrane permeability revisited. Microbiol. Rev..

[20] Mann C.M., Cox S.D. (2000). The outer membrane of *Pseudomonas aeruginosa* NCTC 6749 contributes to its tolerance to the essential oil of *Melaleuca alternifolia* (tea tree oil). Lett. Appl. Microbiol..

[21] Sikkema J., de Bont J.A. (1994). Interactions of cyclic hydrocarbons with biological membranes. J. Biol. Chem..

[22] Tiwari B.K., Valdramidis V.P. (2009). Application of natural antimicrobials for food preservation. J. Agric. Food Chem..

[23] Marrufo T., Nazzaro F. (2013). Chemical composition and biological activity of the essential oil from leaves of *Moringa oleifera* Lam. cultivated in mozambique. Molecules.

[24] Afroditi S., Constantina N. (1997). Antimicrobial, cytotoxic, and antiviral activities of *Salvia fruticosa* essential oil. J. Agric. Food Chem..

[25] Rajagopala S.V., Sikorski P. (2014). The binary protein-protein interaction landscape of *Escherichia coli*. Nat. Biotechnol..

[26] Miyazawa M., Shindo M. (2002). Metabolism of (+)- and (−)-limonenes to respective carveols and perillyl alcohols by cyp2c9 and cyp2c19 in human liver microsomes. Drug Metab. Dispos..

[27] Da F.C., Simão M. (2011). Efficacy of monoterpene perillyl alcohol upon survival rate of patients with recurrent glioblastoma. J. Cancer Res. Clin..

[28] De Saldanha da Gama Fischer J., Costa Carvalho P., da Fonseca C.O., Liao L., Degrave W.M., da Gloria da Costa Carvalho M., Yates J.R., Domont G.B. (2011). Chemo-resistant protein expression pattern of glioblastoma cells (a172) to perillyl alcohol. J. Proteome Res..

[29] Aisa H.A., Lin J. (2012). Chemical Composition, Antimicrobial and Antitumor Activities of the Essential Oils and Crude Extracts of *Euphorbia macrorrhiza*. Molecules.

[30] Luo M., Qiu J. (2012). α-cyperone alleviates lung cell injury caused by *Staphylococcus aureus* via attenuation of α-hemolysin expression. J. Microbiol. Biotechnol..

[31] Zhang L.Y., Lv S., Wu S.C., Guo X., Xia F., Hu X.R., Song Z., Zhang C., Qin Q.Q., Fu B.D. (2014). Inhibitory effects of α-cyperone on adherence and invasion of avian pathogenic *Escherichia coli*, o78 to chicken type ii pneumocytes. Vet. Immunol. Immunopathol..

[32] Jung S.H., Su J.K. (2013). α-Cyperone, isolated from the rhizomes of cyperus rotundus, inhibits LPS-induced cox-2 expression and PGE_2_, production through the negative regulation of NFκB signalling in RAW 264 7 cells. J. Ethnopharmacol..

[33] Lee B.K., Kim J.H. (2005). Myristicin-induced neurotoxicity in human neuroblastoma sk-n-sh cells. Toxicol. Lett..

[34] Chinese Pharmacopoeia Commission (2005). Pharmacopoeia of the People’s Republic of China.

[35] Yamaguchi T., Takamura H. (1998). HPLC method for evaluation of the free radical-scavenging activity of foods by using 1,1-diphenyl-2-picrylhydrazyl. Biosci. Biotechnol. Biochem..

[36] Omp S., Tejk B. (2009). DPPH antioxidant assay revisited. Food Chem..

[37] Cockerill F.R. (2012). Methods for Dilution Antimicrobial Susceptibility Tests for Bacteria that Grow Aerobically.

[38] Işcan G., Kirimer N. (2002). Antimicrobial screening of *Mentha piperita* essential oils. J. Agric. Food Chem..

[39] De M.L., De F.V. (2009). Chemical composition and antimicrobial activity of the essential oils from three chemotypes of *Origanum vulgare* L. ssp. *hirtum* (Link) Ietswaart growing wild in Campania (Southern Italy). Molecules.

[40] Junru H.U., Jiang H. (2013). Study on three non-water-soluble solvents influencing of inhibitory activity in antibacterial test. West. J. Tradit. Chin. Med..

[41] Silva S.L.D., Figueiredo P.M., Yano T. (2007). Cytotoxic evaluation of essential oil from *Zanthoxylum rhoifolium* Lam. leaves. Acta Amazon..

[42] Ghribi L., Nejma A.B. (2016). Chemical composition, cytotoxic and antibacterial activities of the essential oil from the *Tunisian ononis angustissima* L. (Fabaceae). J. Oleo Sci..

